# Global research and emerging trends in autophagy in lung cancer: a bibliometric and visualized study from 2013 to 2022

**DOI:** 10.3389/fphar.2024.1352422

**Published:** 2024-02-27

**Authors:** Bo-Na Liu, Juan Chen, Ying Piao

**Affiliations:** Department of Oncology, General Hospital of Northern Theater Command, Shenyang, China

**Keywords:** autophagy, bibliometrics, lung cancer, VOSviewer, citespace

## Abstract

**Purpose:** To highlight the knowledge structure and evolutionary trends in research on autophagy in lung cancer.

**Methods:** Research publications on autophagy in lung cancer were retrieved from the Web of Science Core Collection database. VOSviewer and CiteSpace data analysis software were used for the bibliometric and visualization analysis of countries, institutions, authors, journals, and keywords related to this field.

**Results:** From 2013 to 2022, research on autophagy in lung cancer developed rapidly, showing rising trends in annual publications and citations. China (1,986 papers; 48,913 citations), Shandong University (77 publications; 1,460 citations), and Wei Zhang (20 publications; 342 citations) were the most productive and influential country, institution, and author, respectively. The journal with the most publications and citations on autophagy in lung cancer was the International Journal of Molecular Sciences (93 publications; 3,948 citations). An analysis of keyword co-occurrence showed that related research topics were divided into five clusters: 1) Mechanisms influencing autophagy in lung cancer and the role of autophagy in lung cancer; 2) Effect of autophagy on the biological behavior of lung cancer; 3) Regulatory mechanisms of 2 cell death processes: autophagy and apoptosis in lung cancer cells; 4) Role of autophagy in lung cancer treatment and drug resistance; and 5) Role of autophagy-related genes in the occurrence and development of lung cancer. Cell proliferation, migration, epithelial-mesenchymal transition, and tumor microenvironment were the latest high-frequency keywords that represented promising future research directions.

**Conclusion:** This is the first comprehensive study describing the knowledge structure and emerging frontiers of research on autophagy in lung cancer from 2013 to 2022 by means of a bibliometric analysis. The study points to promising future research directions focusing on in-depth autophagy mechanisms, clinical applications, and potential therapeutic strategies, providing a valuable reference for researchers in the field.

**Systematic Review Registration**: [https://systematicreview.gov/], identifier [registration number].

## 1 Introduction

Lung cancer is one of the most common cancers and the leading cause of cancer-related death worldwide (Siegel et al., 2023). In 2023, it is projected that there will be approximately 238,340 new cases and 127,070 deaths attributed to lung and bronchus cancer in the United States (Siegel et al., 2023). Unfortunately, the incidence of lung cancer shows a persistent annual increase (Sung et al., 2021; Oliver, 2022). Non-small cell lung cancer (NSCLC) is the most common histological subtype, accounting for approximately 85% of all cases (Zappa and Mousa, 2016; Hendriks et al., 2023). Based on cell type and location, NSCLC can be divided into three main types: adenocarcinoma, squamous cell carcinoma, and large cell carcinoma (Viktorsson et al., 2014; Khilwani and Singh, 2023). Globally, the 5-year survival rate for lung cancer is less than 17% (Nicholson et al., 2022; Siegel et al., 2023). In essence, lung cancer exerts a considerable influence on global quality of life, resulting in significant socioeconomic burdens. A comprehensive understanding of the fundamental mechanisms driving the initiation and progression of lung cancer is imperative for effective prevention and treatment strategies.

Autophagy is a cellular catabolic mechanism responsible for the degradation and recycling of damaged organelles and excessive or abnormal proteins in lysosomes (Mizushima and Komatsu, 2011). In mammals, autophagy can be broadly classified into three main categories based on the nature of the cargo and the mechanisms of cargo delivery to lysosomes: macroautophagy, microautophagy, and chaperone-mediated autophagy (Mizushima, 2018). Autophagy is an evolutionarily conserved process that, under physiological conditions, is generally maintained at low levels to preserve cellular, tissue, and organismal homeostasis while regulating physiological functions (Dikic and Elazar, 2018). Dysregulation of autophagy is implicated in various human diseases, encompassing neurodegenerative diseases, immune disorders, inflammatory diseases, and cancer (Levine and Kroemer, 2019). Recent research has significantly underscored the multifaceted roles of autophagy in different cancers. Autophagy emerges as a pivotal regulatory factor in malignant development, influencing processes such as proliferation, drug resistance, invasion, and metastasis in lung cancer and various other tumors.

The bibliometric analysis of scientific research literature is a systematic and quantitative approach. It involves in-depth examination of publications using techniques such as co-word analysis, collaboration network analysis, and clustering analysis (Ninkov et al., 2022). The goal of this method is to summarize the development of specific research topics, revealing hotspots, emerging trends, and contributions through quantitative statistics on authors, journals, research institutions, or countries within the literature. Numerous bibliometric studies have already focused on investigating literature in the fields of clinical medicine and biomedical research (Wang et al., 2019; Kokol et al., 2021; Peng et al., 2022).

Autophagy functions as a stress response, being inducible in lung cells and intricately associated with the biochemical characteristics of these cells. This response becomes apparent under distinct conditions, including hypoxia, exposure to particles and cigarette smoke, pro-inflammatory states, and the induction of endoplasmic reticulum (ER) stress or oxidative stress. Studies have identified that in hypoxic regions within certain tumors, autophagy is upregulated to mitigate tumor-associated inflammation and furnish the necessary energy supply for cancer cell survival (Degenhardt et al., 2006; Amaravadi et al., 2016; Yazdani et al., 2020). Autophagy is activated in the late stages of malignant tumors and supports tumor cell growth by providing energy from digested molecules (White, 2015; Leonardi et al., 2019). Research on the role of autophagy in lung cancer has made significant advancements. It has been revealed that lung cancer cells rely on substrates to meet their energy needs and autophagy helps provide an adequate energy supply (Guo et al., 2016). However, a comprehensive visual analysis of the research focus and trends in this field is currently absent. Therefore, we utilized various bibliometric tools to conduct a thorough analysis of autophagy-related research in lung cancer from 2013 to 2022. Our objective was to provide researchers with fresh insights and perspectives in the field of autophagy and lung cancer by summarizing the global knowledge structure and pinpointing evolving trends.

## 2 Materials and methods

### 2.1 Data source

The Web of Science Core Collection (WoSCC) database was chosen as the data source for bibliometric analysis due to its widespread recognition in previous research. We downloaded relevant publication data from 2013 to 2022 in “plain text” format.

### 2.2 Data collection

**FIGURE 1:**
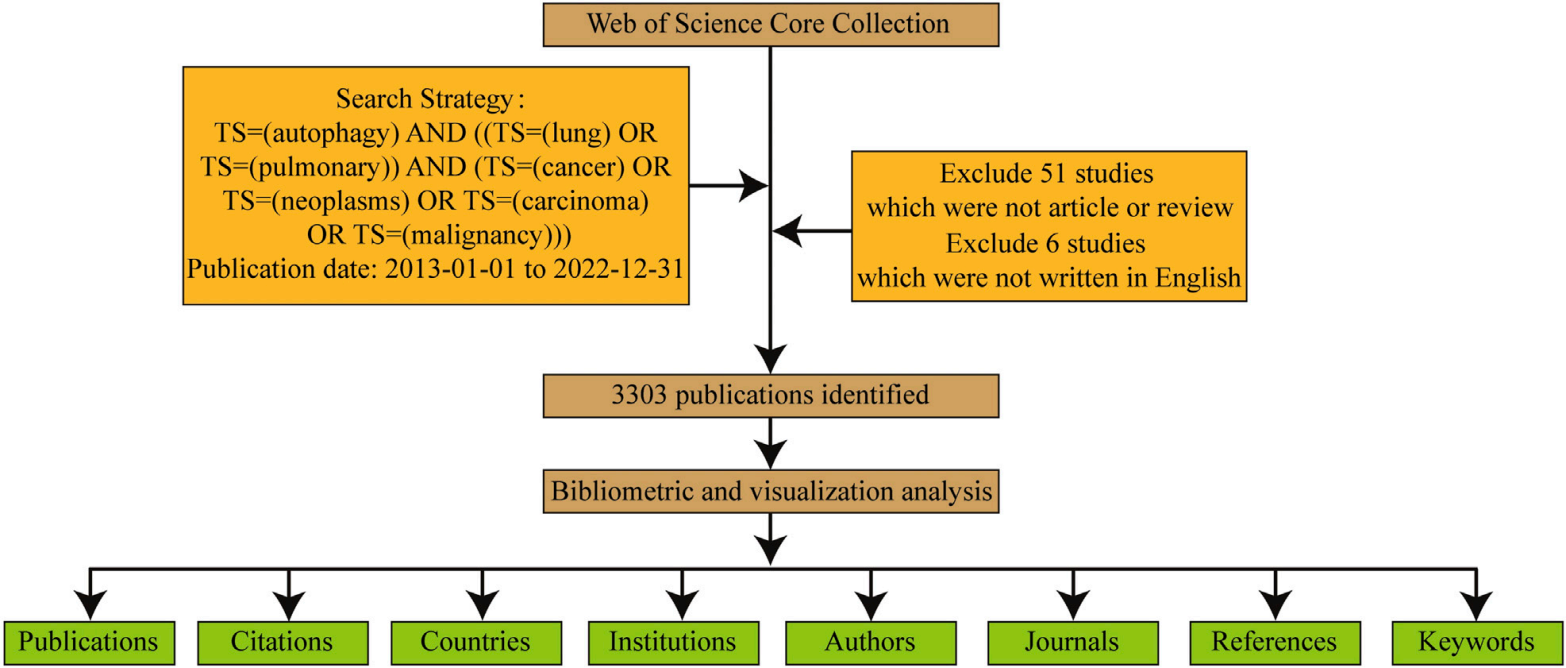
The data collection and retrieval strategy.

Data collection and retrieval strategies are depicted in Figure 1. Publications were required to meet the following criteria.

(1) Retrieval strategy included: The search terms were determined by the TS (“topic,” including title, abstract, author’s keywords and keywords Plus) as TS = (“cancer” OR “neoplasms” OR “carcinoma” OR “malignancy”) AND TS = (“lung” OR “pulmonary”) AND TS=(“autophagy”)
(2) Publication type limited to “article” or “review”;
(3) Publication date ranging from 2013 to 2022;
(4) Publication language limited to English only;
(5) Collected information encompassed publication details, authors, countries, institutions, journals, keywords, and citations.

The two authors (BN L and J C) independently screened the search results by reviewing titles and abstracts, and when necessary, reading the full text. Papers unrelated to autophagy in lung cancer were excluded. Any divergent opinions were resolved through consultation or, if needed, reviewed by an experienced corresponding author (Y P).

### 2.3 Bibliometric and visualized analysis

The bibliometric analysis and visualization of publications retrieved from WoSCC were conducted using VOSviewer, CiteSpace, and GraphPad Prism data analysis software.

VOSviewer, a literature knowledge visualization software based on similarity visualization technology, was chosen due to its ability to generate more well-structured mappings compared to other commonly used bibliometric software (van Eck and Waltman, 2010). In this study, VOSviewer was employed to perform co-occurrence analysis on autophagy-related research in lung cancer, facilitating the establishment of cooperation network maps among countries, institutions, and authors, as well as keyword network maps.

CiteSpace, a prominent software tool for visualizing literature knowledge, was employed in this study to analyze and visualize autophagy-related research in lung cancer. By examining the knowledge structure within this field and identifying current research trends, CiteSpace facilitated the prediction of developmental trajectories and provided insights into research hotspots and development processes (Synnestvedt et al., 2005).

Additionally, GraphPad Prism were employed for the statistical analysis of the distribution of publication outputs over time and space.

## 3 Results

### 3.1 Annual publication trends

**FIGURE 2:**
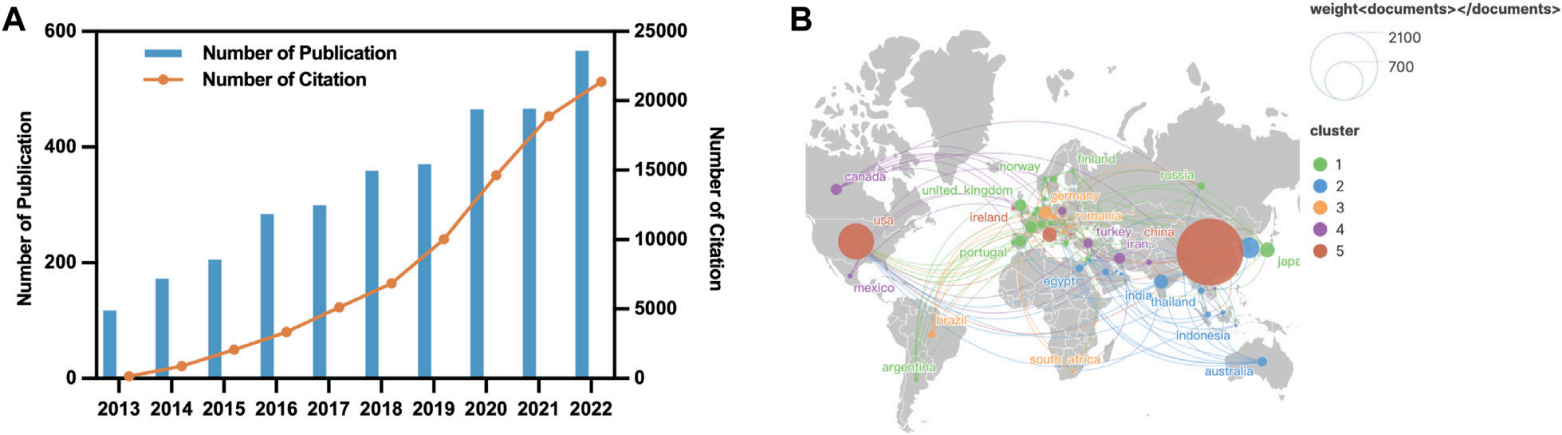
Geographic and temporal distribution of publications. **(A)** Annual publication and citation between 2013–2022. **(B)** Distribution and co-authorship network of countries/regions.

A total of 3,303 studies from 2013 to 2022 related to autophagy in lung cancer were retrieved from WoSCC. Figure 2A depicts the annual publication trends of publications in this domain. Notably, over the past decade, a steady increase has occurred in the number of research articles on autophagy in lung cancer. Global annual publications have risen from 117 in 2013 to 566 in 2022. Furthermore, the annual citation count has consistently shown an upward trajectory throughout the years.

### 3.2 Publication output and collaboration distribution of countries

Research on autophagy in lung cancer is a globally recognized subject, with contributions from 80 countries, as depicted in Figure 2B. Table 1 outlines the top 10 countries in terms of productivity. China (1,986 papers; 48,913 citations) emerges as the most productive country, followed by the United States (583 papers; 31,266 citations) and South Korea (193 papers; 3,978 citations). However, the average citation per article for China (24.63 citations) falls behind that of countries such as France (57.7 citations), the United States (53.63 citations), and Germany (49.01).

**TABLE 1:** The top 10 productive countries/regions.

Rank	Country	Publications	Citations	Average citations
1	China	1986	48,913	24.63
2	United States	583	31,266	53.63
3	South Korea	193	3,978	20.61
4	Japan	98	3,290	33.57
5	Italy	95	4,514	47.52
6	India	92	2,531	27.51
7	Germany	72	3,529	49.01
8	France	63	3,635	57.70
9	United Kingdom	63	2,831	44.94
10	Spain	61	1826	29.93

This study employed VOSviewer to conduct a collaboration analysis among countries, aiming to unveil the international collaboration landscape in this field. With a minimum number of publications for inclusion set at five, a total of 50 countries met the criteria, forming a collaborative network with five clusters represented by distinct colors (Figure 2B). The United States led in the number of collaborative partners (n = 46), followed by China (n = 39), Germany (n = 36), Italy (n = 34), and the United Kingdom (n = 34).

### 3.3 Publication output and collaboration distribution of institutions

A total of 3,129 institutions have contributed to research on autophagy in lung cancer. Table 2 presents the top 20 institutions in terms of productivity. Shandong University (77 publications; 1,460 citations) emerges as the most productive institution, followed by Fudan University (75 publications; 2,487 citations), and the Chinese Academy of Sciences (72 publications; 2,296 citations). With a minimum publication requirement set at 10, a total of 139 institutions met the criteria.

**TABLE 2:** The top 20 productive institutions.

Rank	Institution	Country	Publications	Citations	Average citations
1	Shandong University	China	77	1,460	18.96
2	Fudan University	China	75	2,487	33.16
3	Chinese Academy of Sciences	China	72	2,296	31.89
4	Nanjing Medical University	China	65	2,259	34.75
5	China Medical University	China	64	2,153	33.64
6	Zhejiang University	China	64	1,275	19.92
7	Shanghai Jiao Tong University	China	63	1826	28.98
8	Sichuan University	China	61	1729	28.34
9	Zhengzhou University	China	58	1,612	27.79
10	Huazhong University of Science and Technology	China	52	1,382	26.58
11	Soochow University	China	49	1,344	27.43
12	Xi’an Jiaotong University	China	48	1,157	24.10
13	Chongqing medical university	China	44	760	17.27
14	Sun Yat-sen University	China	44	1,565	35.57
15	Guangzhou Medical University	China	41	1,488	36.29
16	Wuhan University	China	41	1,166	28.44
17	University of Texas MD Anderson Cancer Center	United States	39	1,227	31.46
18	Southern Medical University	China	37	725	19.59
19	Taipei Medical University	China	37	793	21.43
20	Virginia Commonwealth University	United States	37	1708	46.16

**FIGURE 3:**
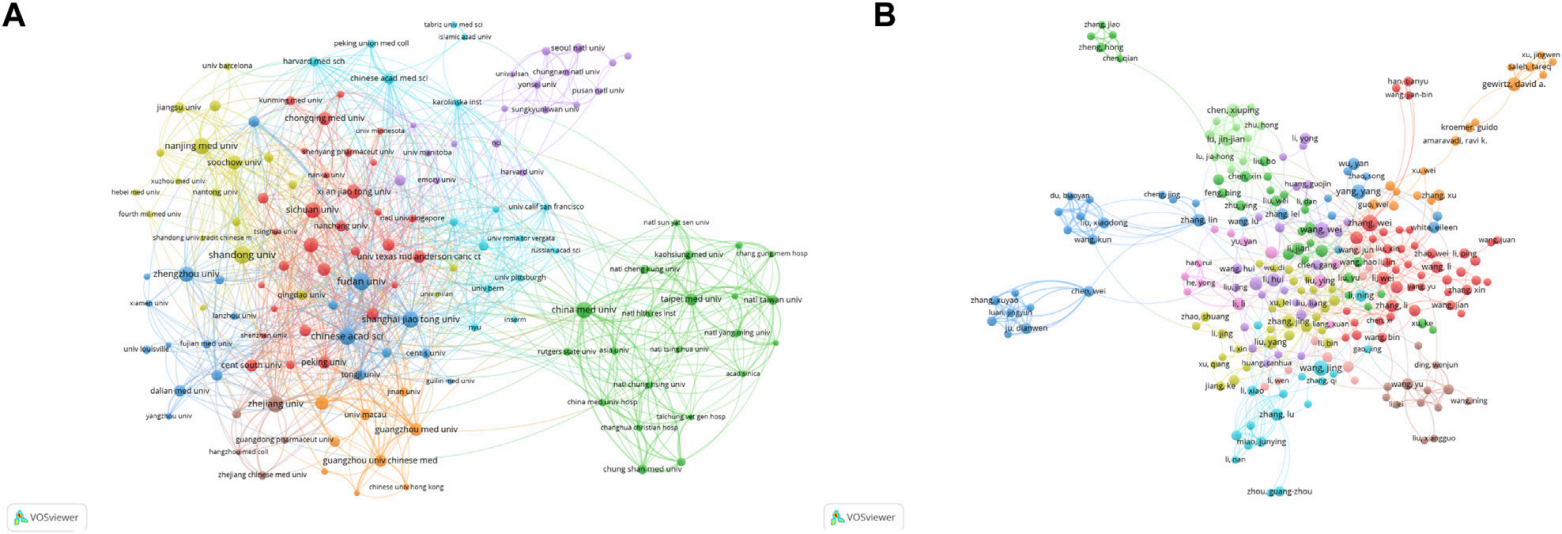
The co-authorship network of institutions **(A)** and authors **(B)**.

Using VOSviewer, a collaboration analysis was conducted on these institutions, and the results are depicted in Figure 3A. The collaboration network comprises eight clusters represented by different colors. The Chinese Academy of Sciences (n = 67), Shanghai Jiao Tong University (n = 41), and Fudan University (n = 35) are positioned at the center of the collaboration network and have the highest number of collaborative partners.

### 3.4 Publication output and collaboration distribution of authors

Between 2000 and 2021, a total of 19,108 authors contributed to research on autophagy in lung cancer. Table 3 presents the top 20 most productive authors. Wei Zhang (20 publications; 342 citations) emerges as the most prolific author, followed by Wei Wang (19 publications; 353 citations) and Jing Wang (17 publications; 512 citations). A co-authorship analysis of the current research field was conducted using VOSviewer. With a minimum publication requirement set at five, a total of 295 authors met the criteria. Among them, 239 authors formed a collaborative network comprising 11 clusters (Figure 3B), with different colors representing clusters. Wei Zhang (n = 19), Wei Li (n = 15), and Lu Zhang (n = 14) have the highest number of collaborative partners and are positioned at the center of the collaboration network.

**TABLE 3:** The top 20 productive authors.

Rank	Author	Publications	Citations	Average citations
1	Zhang, Wei	20	342	17.10
2	Wang, Wei	19	353	18.58
3	Wang, Jing	17	512	30.12
4	Yang, Yang	16	767	47.94
5	Gewirtz, David A	15	849	56.60
6	Liu, Yang	14	571	40.79
7	Chen, Xiuping	14	462	33.00
8	Zhang, Jing	14	404	28.86
9	Liu, Ying	14	379	27.07
10	Li, Hui	14	262	18.71
11	Li, Li	13	354	27.23
12	Li, Wei	13	354	27.23
13	Wang, Lei	12	456	38.00
14	Zhang, Lin	12	450	37.50
15	Li, Yi	12	331	27.58
16	Li, Jian	12	317	26.42
17	Zhang, Li	11	342	31.09
18	Lu, Jin-Jian	11	304	27.64
19	Wu, Yan	11	258	23.45
20	Li, Yan	11	252	22.91

### 3.5 Source journal distribution

VOSviewer was utilized to analyze the source journals of publications. Table 4 lists the top 20 journals that published the most relevant articles, with a publication count of 25 or more. Among these, the journal with the highest publication and citation counts was *International Journal of Molecular Sciences* (93 papers; 3,948 citations). Of the top 20 journals in terms of publication count, the journals with the highest impact factor were *Autophagy* (IF 13.3), *Cancer Letters* (IF 9.7), and *Cell Death and Disease* (IF 9.0).

**TABLE 4:** The top 20 productive journals.

Rank	Journal	If 2022	JCR quartile 2022	Publications	Citations
1	International Journal Of Molecular Sciences	5.6	Q2/Q2	93	3,948
2	Cell Death and Disease	9	Q1	73	2,621
3	Cancers	5.2	Q2	68	1,280
4	Frontiers In Oncology	4.7	Q2	61	965
5	Scientific Reports	4.6	Q1	59	1845
6	Oncology Reports	4.2	Q2	57	1,300
7	Oncology Letters	2.9	Q3	56	1,007
8	Frontiers In Pharmacology	5.6	Q1	55	1,069
9	Biomedicine and Pharmacotherapy	7.5	Q1/Q1	53	1,688
10	Plos One	3.7	Q1	52	1739
11	Biochemical And Biophysical Research Communications	3.1	Q2/Q2	49	873
12	Autophagy	13.3	Q1	46	2,705
13	Oncotargets And Therapy	4	Q2/Q2	40	1,013
14	International Journal Of Oncology	5.2	Q1	33	954
15	Molecular Medicine Reports	3.4	Q2/Q2	32	658
16	Journal Of Cellular Physiology	5.6	Q1/Q1	29	868
17	Cancer Letters	9.7	Q1	28	1,484
18	Molecules	4.6	Q2/Q3	28	586
19	Cancer Cell International	5.8	Q1	27	607
20	Life Sciences	6.1	Q1/Q1	25	431

**FIGURE 4:**
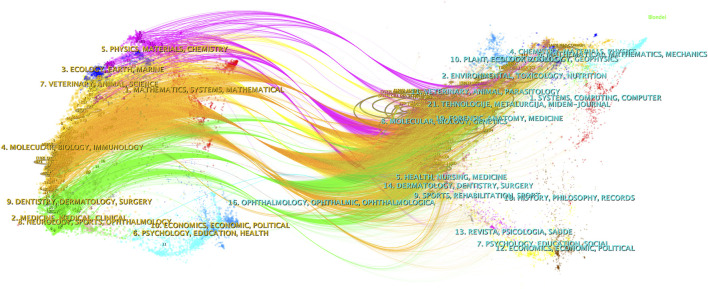
Dual-map overlay of journals related to research on autophagy in lung cancer.

CiteSpace was employed to analyze the citation status between journals publishing relevant studies, and a dual-map overlay was presented (Figure 4). The left half of the figure represents the citing journals while the right half represents the cited journals. The curved line from left to right represents a citation-path connecting line, which demonstrates the knowledge flow and connection in different research fields. As shown in Figure 4, research on autophagy in lung cancer is mainly distributed in disciplines such as Molecular, Biology, Immunology, Medicine, Medical, and Clinical, and its citing literature is also mainly from these disciplines.

### 3.6 Most cited and co-cited articles

Using VOSviewer, citation and co-citation analyses were conducted in this research field. Over the past 10 years, 47 articles in this field have been cited over 200 times, with a total of 139,314 references cited in the publications. Among these, 71 articles have been cited over 50 times. Table 5 and Table 6 list the articles with the highest citation counts and co-citation counts, respectively.

**TABLE 5:** The Top 10 highly cited publications.

Rank	Title	Journal	First author	Year	Citations
1	From Krebs to clinic: glutamine metabolism to cancer therapy	Nat Rev Cancer	Brian J Altman	2016	1,091
2	Histone Deacetylase Inhibitors as Anticancer Drugs	Int J Mol Sci	Tomas Eckschlager	2017	747
3	Cancer-associated cachexia	Nat Rev Dis Primers	Vickie E Baracos	2018	706
4	Emerging functions of the EGFR in cancer	Mol Oncol	Sara Sigismund	1,018	686
5	Reactive oxygen species and cancer paradox: To promote or to suppress?	Free Radic Biol Med	Sehamuddin Galadari	2017	596
6	Recent insights into the function of autophagy in cancer	Genes Dev	Ravi Amaravadi	2016	556
7	Pharmacological modulation of autophagy: therapeutic potential and persisting obstacles	Nat Rev Drug Discov	Lorenzo Galluzzi	2017	529
8	The Roles of Autophagy in Cancer	Int J Mol Sci	Chul Won Yun	2018	504
9	Autophagy, Metabolism, and Cancer	Clin Cancer Res	Eileen White	2015	468
10	Autophagy suppresses progression of K-ras-induced lung tumors to oncocytomas and maintains lipid homeostasis	Genes Dev	Jessie Yanxiang Guo	2013	456

**TABLE 6:** The Top 10 highly co-cited publications.

Rank	Title	Journal	First author	Year	Citations
1	Cancer Statistics, 2017	CA Cancer J Clin	Rebecca L Siegel	2017	189
2	Autophagy in the pathogenesis of disease	Cell	Beth Levine	2008	184
3	Global Cancer Statistics 2020: GLOBOCAN Estimates of Incidence and Mortality Worldwide for 36 Cancers in 185 Countries	CA Cancer J Clin	Hyuna Sung	2021	178
4	Hallmarks of cancer: the next-generation	Cell	Douglas Hanahan	2011	166
5	LC3, a mammalian homologue of yeast Apg8p, is localized in autophagosome membranes after processing	EBMO J	Y Kabeya	2000	143
6	Deconvoluting the context-dependent role for autophagy in cancer	Nat Rev Cancer	Eileen White	2012	137
7	AMPK and mTOR regulate autophagy through direct phosphorylation of Ulk1	Nat Cell Biol	Joungmok Kim	2011	136
8	Targeting autophagy in cancer	Nat Rev Cancer	Jean M Mulcahy Levy	2017	134
9	Autophagy fights disease through cellular self-digestion	Nature	Noboru Mizushima	2008	123
10	Methods in mammalian autophagy research	Cell	Noboru Mizushima	2010	123

### 3.7 Keyword co-occurrence analysis

**FIGURE 5:**
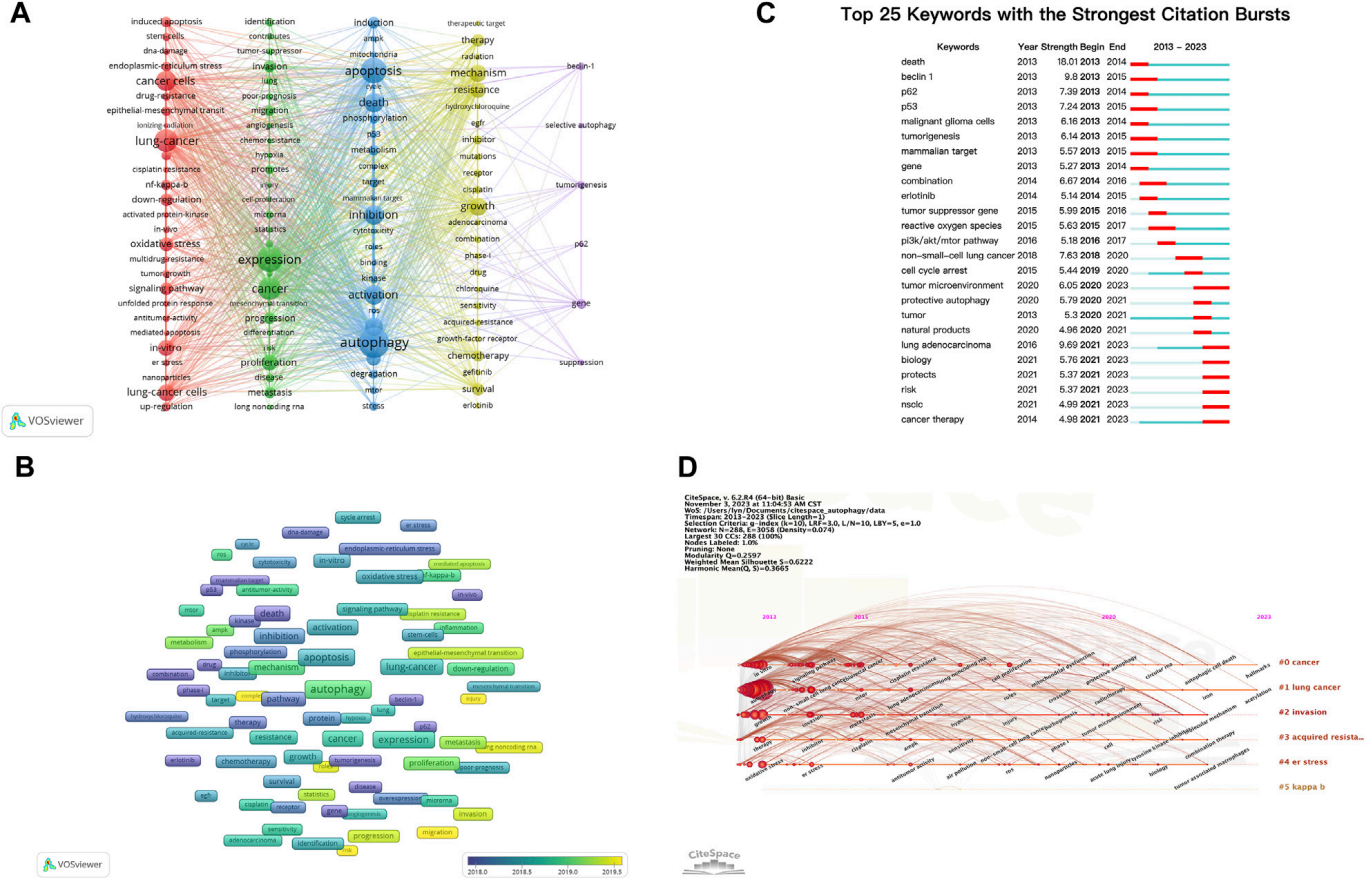
Analysis of dominant keywords. **(A)** The co-occurrence cluster analysis of the top 109 keywords. **(B)** The overlay map of the top 109 keywords. **(C)** Detection of top 25 keywords for the citation burst. **(D)** Timeline view for co-words analysis.

Keywords encompass the main themes of publications, making high-frequency keywords suitable for co-occurrence analysis. In this study, VOSviewer was used to extract and cluster keywords. When the minimum occurrence frequency was set at 30, 109 keywords met the criteria. Table 7 presents the top 100 keywords along with their frequencies and cluster affiliations. Figure 5A displays the network graph of the top 109 keywords and their co-occurrence. Based on the level of similarity, VOSviewer automatically divided the keywords into five clusters, represented by different colors: red (Cluster 1), green (Cluster 2), blue (Cluster 3), yellow (Cluster 4), and purple (Cluster 5). Cluster 1 includes 27 keywords with central keywords such as “lung cancer” (frequency n = 574), “cancer cells” (n = 448), and “oxidative stress” (n = 213). Cluster 2 includes 27 keywords with central keywords such as “expression” (n = 693), “proliferation” (n = 281), and “invasion” (n = 155). Cluster 3 includes 25 keywords with central keywords such as “autophagy” (n = 979), “apoptosis” (n = 718), and “death” (n = 408). Cluster 4 includes 24 keywords with central keywords such as “growth” (n = 364), “mechanism” (n = 323), and “resistance” (n = 265). Cluster 5 includes six keywords with central keywords such as “gene” (n = 110), “tumorigenesis” (n = 71), and “beclin-1" (n = 70).

**TABLE 7:** Clusters of the top 100 Keywords.

Rank	Keywords	Cluster	Counts	Rank	Keywords	Cluster	Counts
1	autophagy	3	979	51	inflammation	2	68
2	apoptosis	3	718	52	p53	3	68
3	expression	2	693	53	receptor	4	66
4	lung-cancer	1	574	54	cisplatin	4	64
5	cancer	2	562	55	lung	2	62
6	cancer cells	1	448	56	microrna	2	62
7	death	3	408	57	mtor	3	62
8	activation	3	403	58	combination	4	61
9	inhibition	3	398	59	tumor-suppressor	2	61
10	growth	4	364	60	antitumor-activity	1	60
11	pathway	3	364	61	drug	4	58
12	mechanism	4	323	62	er stress	1	58
13	lung-cancer cells	1	310	63	mutations	4	57
14	proliferation	2	281	64	poor-prognosis	2	55
15	*in vitro*	1	273	65	acquired-resistance	4	52
16	resistance	4	265	66	dna-damage	1	52
17	protein	3	244	67	*in vivo*	1	52
18	oxidative stress	1	213	68	sensitivity	4	52
19	signaling pathway	1	180	69	mitochondria	3	50
20	survival	4	171	70	overexpression	2	50
21	downregulation	1	167	71	phase-i	4	50
22	therapy	4	167	72	ampk	3	47
23	invasion	2	155	73	contributes	2	47
24	induction	3	154	74	activated protein-kinase	1	45
25	progression	2	148	75	multidrug-resistance	1	45
26	chemotherapy	4	144	76	adenocarcinoma	4	44
27	metastasis	2	143	77	cisplatin resistance	1	44
28	induced apoptosis	1	128	78	growth-factor receptor	4	44
29	nf-kappa-b	1	125	79	suppression	5	44
30	metabolism	3	124	80	radiation	4	43
31	migration	2	116	81	unfolded protein response	1	41
32	phosphorylation	3	111	82	gefitinib	4	40
33	target	3	111	83	ros	3	40
34	gene	5	110	84	chloroquine	4	39
35	epithelial-mesenchymal transition	1	109	85	egfr	4	39
36	endoplasmic-reticulum stress	1	107	86	nanoparticles	1	39
37	cycle arrest	1	99	87	cytotoxicity	3	38
38	promotes	2	98	88	roles	3	38
39	degradation	3	97	89	chemoresistance	2	35
40	stress	3	97	90	erlotinib	4	35
41	upregulation	1	94	91	long noncoding rna	2	35
42	inhibitor	4	91	92	differentiation	2	34
43	stem-cells	1	83	93	p62	5	34
44	drug-resistance	1	78	94	statistics	2	34
45	identification	2	77	95	angiogenesis	2	33
46	kinase	3	77	96	binding	3	33
47	disease	2	73	97	complex	3	33
48	tumor-growth	1	71	98	hypoxia	2	33
49	tumorigenesis	5	71	99	mediated apoptosis	1	33
50	beclin-1	5	70	100	selective autophagy	5	33

To explore the temporal evolution of research hotspots, VOSviewer was used to analyze the average appearance year (AAY) of the top 109 keywords, presented in the form of a heatmap (Figure 5B). We found that earlier appearing keywords include “p62”(AAY: 2016.91), “beclin-1" (AAY: 2017.01), and “radiation” (AAY: 2017.05). The most recently appearing high-frequency keywords include “cell-proliferation” (AAY: 2020.53), “injury” (AAY: 2020.00), and “migration” (AAY: 2019.76).

### 3.8 Keyword burst and timeline view

Keyword burst analysis reflects the degree of acceptance and dissemination of major research topics. CiteSpace was employed to analyze the keyword burst in autophagy-related research in lung cancer from 2013 to 2022. Figure 5C illustrates the top 25 keywords with the highest burst intensity. Keywords with the strongest burst intensity include “death” (Strength = 18.01), “beclin 1"(Strength = 9.80), and “lung adenocarcinoma” (Strength = 9.69), indicating the widespread acceptance of these topics. Recent keywords in a burst state include “cancer therapy” (2021–2023), “NSCLC” (2021–2023), “risk” (2021–2023), and “tumor microenvironment (TME)" (2020–2023), indicating the current hotspots in these areas.

The keyword timeline view describes the trend of changing research hotspots over time in a field. CiteSpace was used to analyze the timeline view of autophagy-related research in lung cancer from 2013 to 2022 (Figure 5D). All keywords were divided into six clusters, with the number of keywords in a cluster indicating the importance of that topic in the field. The ranking of clusters is determined by the number of citations. Clusters #0 and #1 almost cover the entire timeline, indicating that they have consistently been key topics in the field.

## 4 Discussion

The utilization of bibliometrics and visualization analysis offers a means to examine the attributes and evolving patterns of literature, encompassing both the literature itself and various indicators associated with it. This investigation endeavors to evaluate the contributions made by countries, institutions, and authors in the realm of autophagy research in lung cancer, along with identifying the fundamental literature, research focal points, and developmental tendencies. The ensuing subsections will delve into a comprehensive discussion of the primary findings derived from this analysis.

Besides, a recent study utilized bibliometric analysis and visualization to examine the role of autophagy in lung diseases (Lin et al., 2022). Although this study shares a common methodological approach with our research in employing bibliometrics to investigate autophagy, there are noticeable differences in their focus, time span, research outcomes, and emphasized content. Firstly, the mentioned study encompasses all lung diseases, including lung cancer, chronic obstructive pulmonary disease (COPD), and COVID-19-related pneumonia, while our study specifically focuses on lung cancer. In comparison, our study may lack breadth by concentrating on a single disease but offers a more in-depth analysis within the context of lung cancer. Secondly, our research covers the time span from 2013 to 2022, providing a more current perspective. Finally, regarding the analysis of research trends, the mentioned study suggests that, over the past decade, most research has involved basic studies on pathways related to the regulatory role of autophagy in suppressing and alleviating lung diseases. In contrast, our study analyzes five clusters of research trends in autophagy in lung cancer, including its impact on cancer mechanisms, biological behaviors, regulation of cell death processes, roles in treatment and drug resistance, and the involvement of autophagy-related genes in the occurrence and development of lung cancer.

### 4.1 Trends and current status of global publications

In the previous decade, the investigation of autophagy in the context of lung cancer has experienced a phase of accelerated advancement, as evidenced by the annual increment in the quantity of relevant publications. Notably, over the course of the last 5 years, the volume of publications has exhibited a twofold increase in comparison to the preceding 5-year period, thereby indicating a sustained and rapid surge in interest within this particular research domain. Consequently, it is plausible to anticipate a more comprehensive and profound exploration of autophagy in the context of lung cancer in the forthcoming years.

Over the past decade, a total of 3,303 papers related to autophagy in lung cancer were published by 80 countries globally. China contributed the most publications (1,986 papers), accounting for 60.13% of the total, followed by the United States (583 papers) and South Korea (193 papers). However, the average citation rate of papers from China (24.63 citations) is lower than that of countries such as France (57.7 citations), the United States (53.63 citations), and Germany (49.01 citations). Of the top 20 institutions in terms of publication quantity, 19 are from China, including Shandong University (77 papers), Fudan University (75 papers), and the Chinese Academy of Sciences (72 papers). The 20th position is occupied by Virginia Commonwealth University (37 papers) from the United States, which has the highest average citation rate among the top 20 institutions, with 46.16 citations per paper. In terms of authors, Wei Zhang (20 papers), Wei Wang (19 papers), and Jing Wang (17 papers) from China have published the most articles in the field of autophagy in lung cancer. Of the top 20 authors in terms of publication quantity, the most cited and with the highest average citation rate is David A. Gewirtz from Virginia Commonwealth University in the United States (849 citations, average citations 56.60). Our findings indicate that research on autophagy in lung cancer has attracted widespread attention worldwide, with China and the United States leading in this field. However, a noticeable imbalance exists between the quantity and quality of papers published by China in this field.

Additionally, this study constructed a collaboration network among countries, institutions, and authors to assess collaboration within this research field. The findings of this investigation have revealed that the United States (n = 46), China (n = 39), and Germany (n = 36) have the most collaboration partners, indicating that authors from these countries prefer international partnerships. Institutions with the highest publication volume are often the ones most inclined to seek collaboration, placing them at the center of various collaboration networks. These include Fudan University, Nanjing Medical University, and China Medical University. Similarly, authors with the highest publication volume are positioned at the center of author collaboration networks. The data suggests that collaboration with other institutions/authors may facilitate the rapid development of research in the field.

This study provides a comprehensive overview of the principal journals that publish research on autophagy in lung cancer. Scholars engaged in this area of study are advised to consider these journals as primary outlets for their related scholarly contributions. Among the top 20 journals with the most publications in this field, 18 journals are categorized as Q1/Q2 in the Journal Citation Reports (JCR). Notably, *AUTOPHAGY* stands out with the highest impact factor (IF) of 13.3, and boasts the highest average citation rate (58.8 citations) among the published articles, underscoring its substantial reference value and academic significance in this research field.

### 4.2 Research hotspots in autophagy-related research in lung cancer

Co-occurrence analysis serves as a valuable tool in the identification of pertinent subjects and trending topics within a particular research domain, thereby providing researchers with valuable insights for potential comprehensive inquiries. By conducting a co-occurrence mapping of keywords within the realm of autophagy-related research in lung cancer, it becomes apparent that certain keywords, namely, autophagy, apoptosis, expression, lung cancer, and death, exhibit significantly larger sizes and stronger relationships compared to other keywords. Furthermore, the extracted keywords from all encompassed studies were systematically classified into five distinct clusters, each corresponding to a specific research topic: Cluster One: Mechanisms influencing autophagy in lung cancer and the role of autophagy in lung cancer; Cluster Two: Effect of autophagy on the biological behavior of lung cancer cells; Cluster Three: Regulatory mechanisms of two types of cell death in lung cancer cells: autophagy and apoptosis; Cluster Four: Role of autophagy in lung cancer treatment and drug resistance; Cluster Five: Role of autophagy-related genes in the occurrence and development of lung cancer.

#### 4.2.1 Cluster one: mechanisms influencing autophagy in lung cancer and the role of autophagy in lung cancer

This cluster encompasses a total of 27 keywords, including lung cancer, oxidative stress, epithelial-mesenchymal transition (EMT), ER stress, and others. The central theme of this cluster can be summarized as the mechanisms influencing autophagy in lung cancer as well as the role of autophagy in lung cancer.

Under physiological conditions, autophagy stands as an evolutionarily conserved process, activated in response to stress or nutrient depletion. This process effectively facilitates intracellular metabolism through the degradation of cellular components within lysosomes, thereby generating vital compounds necessary to fulfill cellular metabolic and energy demands (Bagherniya et al., 2018; He et al., 2018; Guo et al., 2022). In the context of tumorigenesis and development, autophagy serves a dual function. First, it mitigates cellular damage, including DNA damage, thereby impeding the formation of tumors. Conversely, once a tumor has formed, autophagy aids in the sustenance of cancer by safeguarding aggressive tumor cells amidst challenging circumstances, thereby facilitating growth (Mukhopadhyay et al., 2022). Autophagy assumes a pivotal role in adapting to the TME, particularly in conditions characterized by oxidative stress and hypoxia. Autophagy regulates energy metabolism, modulates tumor-associated fibroblasts, mediates angiogenesis and invasion, promotes immunological responses, maintains cancer stem cell survival, induces chemotherapy resistance, and contributes to treatment relapse (Mowers et al., 2018; Camuzard et al., 2020; Mukhopadhyay et al., 2022).

The interconnection between autophagy and oxidative stress is evident within cellular processes. Oxidative stress refers to the excessive accumulation of reactive oxygen species (ROS) within cells, triggering redox reactions, disrupting cellular biomolecules, and causing cell damage. Under pathological conditions, excess ROS can induce upregulation of the autophagy pathway to clear damaged organelles and waste proteins within the ER lumen. Under oxidative stress conditions, autophagy helps cells eliminate ROS and other harmful substances, thereby alleviating oxidative stress damage to cells. Furthermore, autophagy can also reduce ROS production by degrading damaged organelles, such as mitochondria, further lowering oxidative stress levels (Filomeni et al., 2015). However, in certain cases, autophagy may increase ROS production instead of clearing ROS. For instance, a nano-hybrid material inducing oxidative stress and triggering autophagy was found to inhibit lung cancer cell growth, suggesting a potential strategy for combined chemotherapy and oxidative stress–induced autophagy in lung cancer treatment (Lu et al., 2015). Furthermore, the activation of CCS, facilitated by H3k27 acetylation, has been shown to alleviate oxidative stress in lung cancer cells, thereby promoting autophagy and impeding cell apoptosis. Consequently, CCS emerges as a promising therapeutic target for lung adenocarcinoma (Hu et al., 2023). Further investigation is necessary to acquire a more comprehensive comprehension of the intricate interactive mechanisms between autophagy and oxidative stress in the context of lung cancer.

The ER is an intracellular organelle that plays a crucial role in protein folding and lipid synthesis. In the event of any disruption to normal physiological processes within the ER, a defensive response called ER stress is triggered to maintain the stability of its internal milieu. Due to inadequate nutrient supply under pathological conditions, protein synthesis in the ER becomes unregulated, leading to the accumulation of unfolded or misfolded proteins within the ER lumen, triggering the initiation of an unfolded protein response (UPR). The UPR further alters the ubiquitin–proteasome pathway or autophagy process. In various malignant tumors, ER stress can effectively induce cellular autophagy because cancer cells need to continuously utilize their organelles to support their growth. Meanwhile, autophagy is essential for resisting ER expansion induced by ER stress, enhancing cell vitality, and preventing non-apoptotic cell death (Senft and Ronai, 2015; Lin et al., 2019; Bhardwaj et al., 2020). Numerous investigations have been dedicated to elucidating novel pathways that specifically target ER stress and autophagy in the context of lung cancer. For instance, interferon-γ, in addition to its direct induction of apoptosis and cell-cycle arrest in malignant human cells, has been observed to induce ER stress. In turn, UPR is activated and autophagy is attenuated, ultimately culminating in the apoptosis of lung cancer cells (Fang et al., 2021). Furthermore, ER stress and autophagy have been found to play integral roles in the apoptotic response of human lung cancer cells to cisplatin treatment (Shi et al., 2016). High-temperature treatment of NSCLC cells can activate ER stress and induce protective autophagy in response to oxidative stress; inhibiting autophagy can enhance apoptosis of lung cancer cells through the ER stress pathway (Xie et al., 2016). The acidic microenvironment in NSCLC cells promotes protective autophagy by activating ER stress, weakening apoptosis in lung cancer cells (Xie et al., 2015). Autophagy inhibition can overcome erlotinib resistance by regulating ER stress–mediated cell apoptosis (Wang et al., 2016). The aforementioned findings present novel approaches for the treatment of lung cancer, highlighting the importance of manipulating the interplay between autophagy and ER stress to augment the efficacy of therapeutic interventions. However, further research is necessary to gain a comprehensive understanding of the intricate mechanisms governing the interplay between ER stress, autophagy, and their impact on lung cancer progression and responses to therapeutic interventions.

Epithelial-mesenchymal transition (EMT) is widely recognized as a significant catalyst in the advancement and dissemination of cancer (Krebs et al., 2017; Saitoh, 2018). The intricate interplay between autophagy and EMT signaling pathways has been extensively investigated. Previous research has demonstrated that EMT signaling pathways can elicit or impede autophagy, while autophagy also plays a role in the initiation and suppression of EMT. Epithelial-mesenchymal transition requires autophagy to support the survival of cancer cells. However, autophagy can prevent the occurrence of EMT and may weaken the acquisition of the EMT phenotype in cancer cells. Therefore, targeting autophagy to inhibit EMT may become a new method of anticancer treatment (Chen et al., 2019). Several studies have focused on the relationship between autophagy and EMT in lung cancer. These include studies on photodynamic therapy, which can induce autophagy to regulate EMT and promote the occurrence and development of lung cancer. An *in vitro* study has revealed that Curcumin-mediated photodynamic therapy (PDT), in combination with autophagy inhibitors, can suppress epithelial-mesenchymal transition (EMT) in lung cancer cells. This holds promise as a prospective strategy for inhibiting invasion and migration in lung cancer (Shao et al., 2022). Through *in vitro* cell experiments and *in vivo* animal experiments, it has been found that CCL2 promotes EMT and metastasis of NSCLC through the PI3K/Akt/mTOR axis and the autophagy signaling pathway (Xu et al., 2023). The NOTCH1 intracellular domain (NICD) serves as a crucial transcriptional regulatory factor in EMT and tumor metastasis. Autophagic activation can stimulate the degradation of NICD, significantly inhibiting the migration and invasion of *in vitro* lung cancer cells (Zada et al., 2022). Alizadeh and others have highlighted the pivotal role of autophagy as a potential positive regulator of transforming growth factor (TGF)β1-induced EMT in vitro NSCLC cells. They have identified autophagy inhibitors as promising new drugs that antagonize the role of TGFβ1 in the clinical progression of NSCLC (Alizadeh et al., 2018). Utilizing *in vitro* lung cancer cells, *in vivo* mouse models of lung adenocarcinoma, and tissue specimens from lung cancer patients, Lei Han and others discovered that microRNA-106a regulates autophagy-related cell death and epithelial-mesenchymal transition (EMT) in lung cancer bone metastasis by targeting TP53INP1 (Han et al., 2021). The aryl hydrocarbon receptor, a ligand-activated transcription factor, inhibits lung cancer migration by regulating EMT through autophagy in vitroNSCLC cells (Tsai et al., 2017). The NF-κB signaling pathway emerges as a crucial regulatory factor between EMT and autophagy. It can upregulate various EMT markers to promote EMT and, through different mechanisms, stimulate or inhibit autophagy. Autophagy activators may be employed to halt NF-κB signal transduction, thereby inhibiting EMT and cancer invasion and metastasis (Huber et al., 2005; Chen et al., 2019). In summary, cellular autophagy and EMT play indispensable roles in the occurrence and progression of lung cancer, posing new challenges for anticancer therapies. Future research should focus on delving into the genetic and molecular mechanisms regulating autophagy and EMT. Additionally, there is an urgent need to identify various transcription factors and novel EMT markers to devise effective anticancer strategies by disrupting tumor metastasis. Current studies have attempted to manipulate EMT by utilizing existing autophagy modulators to intervene in tumor progression. However, due to inevitable challenges such as high cytotoxicity and low specificity, the clinical application of these approaches remains limited. Therefore, concerted efforts should be made to explore more potential and precise methods to stimulate or inhibit autophagy, thereby restraining EMT and effectively controlling cancer development, especially when combined with other anticancer strategies, offering a more promising therapeutic approach.

#### 4.2.2 Cluster two: effect of autophagy on the biological behavior of lung cancer cells

This cluster encompasses a total of 27 keywords, including expression, proliferation, invasion, progression, metastasis, and others. The central focus of this cluster revolves around the effect of autophagy on the biological behavior of lung cancer cells.

The intricate and diverse influence of autophagy on the biological behavior of lung cancer encompasses various aspects. Autophagy contributes to the preservation of cellular stability through the clearance of damaged organelles and protein waste. Additionally, it actively participates in the TME, regulating immune responses and inflammation. Consequently, autophagy affects multiple biological behaviors of lung cancer cells, such as proliferation, migration, metastasis, and angiogenesis. The close association between autophagy and the occurrence, development, and prognosis of lung cancer necessitates a comprehensive exploration of the underlying mechanisms.

In a study conducted by Strohecker and others, the effect of autophagy deficiency on lung cancer was investigated using a BrafV600E-induced lung cancer mouse model, both in the presence and absence of the tumor suppressor, Trp53. They achieved this by deleting the essential autophagy gene, *Atg7*. Their research revealed that autophagy deficiency may lead to an increase in oxidative stress, thereby accelerating tumor cell proliferation. However, in the later stages of tumor development, the absence of autophagy may result in the accumulation of mitochondrial defects, ultimately suppressing tumor growth (Strohecker et al., 2013). Various genes and signaling pathways, such as PARP1-mediated AMPK-mTOR, RPL11, COTE-1, PINK1, PEDF, and MALAT1, have been identified to play critical roles in regulating autophagy and influencing lung cancer cell proliferation, migration, and invasion (Ma et al., 2018; Lu et al., 2020; Miao et al., 2022; Chen et al., 2023; Ma et al., 2023; Zhang et al., 2023). Additionally, certain compounds, such as the metal, Cd, schizandrin A, pinocembrin, adenine aldehyde, and inhibitors of basal autophagy, have been found to induce autophagy in lung cancer cells, affecting proliferation and migration (Kaminskyy et al., 2012; Wang et al., 2020; Gong, 2021; Zhu et al., 2021; Ghemrawi et al., 2023).

In conclusion, the effect of autophagy on lung cancer is complex and multifaceted, potentially affecting numerous processes, such as cell proliferation, invasion, and migration, in multiple ways. Consequently, conducting comprehensive research in this area is crucial for the development of novel therapeutic strategies within the field.

#### 4.2.3 Cluster three: regulatory mechanisms of two types of cell death in lung cancer cells: Autophagy and apoptosis

This cluster contains a total of 24 keywords, including autophagy, apoptosis, death, activation, inhibition, and others, focusing on the regulatory mechanisms of two types of cell death, autophagy, and apoptosis, in lung cancer cells.

Cell apoptosis is a genetically controlled, autonomous, programmed cell death mechanism crucial for maintaining cellular environmental stability. The relationship between autophagy and apoptosis in lung cancer cells is intricate. Autophagy can prevent the excessive degradation of proteins in tumor cells undergoing starvation or stress. However, sustained activation of autophagy can lead to cell apoptosis or other forms of programmed cell death (White, 2015; Liu et al., 2017). Abundant research suggests that autophagy itself may serve as a mechanism of cell death, referred to as autophagic cell death (ACD), further confirming the potential association of autophagy with the apoptotic process (Marino et al., 2014; Denton and Kumar, 2019). Characteristics of ACD include chromatin condensation, cytoplasmic vacuole accumulation, LC3 lipidation, and caspase-dependent apoptosis. These can be inhibited through the knockout of core autophagy genes or the use of autophagy inhibitors (Marino et al., 2014; Liu et al., 2017). The interconnection between autophagy-related cell death and apoptosis is intricate and multifaceted. The involvement of caspases in autophagy and the role of ATGs in apoptosis further contribute to the intricate overlap of these two biological processes. Consequently, cellular autophagy can function as both a regulator and an executor of cell apoptosis, contingent upon the surrounding microenvironment, treatment interventions, and the developmental stage of the tumor (Liu et al., 2017).

#### 4.2.4 Cluster four: role of autophagy in lung cancer treatment and drug resistance

This cluster comprises a total of 25 keywords, including resistance, chemotherapy, epidermal growth factor receptor (EGFR), gefitinib, erlotinib, and others, effectively summarizing the overarching theme of investigating the role of autophagy in lung cancer treatment and drug resistance.

Autophagy is an intracellular degradation and recycling mechanism that helps cells cope with various environmental stresses and, to some extent, participates in the regulation of cell death. During chemotherapy and radiotherapy, lung cancer cells often employ autophagy as a mechanism to mitigate intracellular damage and stress, thereby enhancing cell survival and evading therapeutic effects (Guo et al., 2022; Wang et al., 2022). Research has demonstrated that lung cancer cells exhibit heightened autophagic activity after prolonged chemotherapy or targeted treatment, resulting in the degradation of drug target proteins and repair-capable proteins. Consequently, the drugs become less effective, leading to the development of drug resistance. In light of the significance of autophagy in lung cancer treatment and resistance, scholars are currently investigating the potential application of autophagy-related regulatory factors or drugs to augment the efficacy of chemotherapy and targeted therapy.

In the case of patients with lung adenocarcinoma treated with tyrosine kinase inhibitors (TKIs), such as erlotinib or gefitinib, targeting the kinase domain of the EGFR, significant clinical improvements have been observed. However, the emergence of resistance remains a critical clinical issue (Nihira et al., 2014). Research by Nihira and others indicates that LC3A-mediated autophagy is involved in the development of resistance to EGFR-TKIs. LC3A stands out as a promising therapeutic target to overcome resistance in lung adenocarcinoma against EGFR-TKIs (Nihira et al., 2014). Studies have shown that in lung cancer cells, autophagy can be activated by EGFR-TKIs, and inhibiting autophagy enhances the growth-inhibitory effects of EGFR-TKIs (Han et al., 2011). Li and others found that erlotinib induces apoptosis and autophagy in NSCLC cells through the activation mutation of EGFR. Inhibition of autophagy enhances the sensitivity of NSCLC cells to erlotinib, suggesting that autophagy may contribute to the emergence of resistance in NSCLC (Li et al., 2013). Lypova and others identified PFKFB3 as a mediator of erlotinib-induced autophagy in NSCLC. Inhibiting PFKFB3, by weakening autophagic flux, sensitizes NSCLCs to erlotinib (Lypova et al., 2021).

In conclusion, an increasing number of studies are focusing on developing combinations of chemotherapy drugs and autophagy inhibitors in lung cancer treatment, Table 8 enumerates the ongoing or completed clinical trials in this field. This provides a theoretical basis for further improving autophagy-based lung cancer treatment strategies.

**TABLE 8:** The Top 10 highly cited publications.

Phase	Title	Conditions	Interventions	Trial ID
II	Modulation of Autophagy in Patients With Advanced/Recurrent NSCLC	NSCLC	Hydroxychloroquine; Paclitaxel; Carboplatin; Bevacizumab	NCT01649947
I/II	Hydroxychloroquine, Carboplatin, Paclitaxel, and Bevacizumab in Recurrent Advanced NSCLC	NSCLC	Hydroxychloroquine; Paclitaxel; Carboplatin; Bevacizumab	NCT00728845
II	Binimetinib and Hydroxychloroquine in Patients With Advanced KRAS Mutant NSCLC	NSCLC	Hydroxychloroquine Pill; Binimetinib Pill	NCT04735068
II	Hydroxychloroquine and erlotinib in NSCLC.	NSCLC	Hydroxychloroquine; Erlotinib	NTR3360
II	Treatment with hydroxychloroquine and erlotinib in patients with NSCLC	NSCLC	Hydroxychloroquine; Erlotinib	EUCTR 2011–004903–20-NL

#### 4.2.5 Cluster five: role of autophagy-related genes in the occurrence and development of lung cancer

This cluster comprises six keywords, including gene, tumorigenesis, beclin-1, p62, and others. The theme of this cluster focuses on the role of autophagy-related genes in the occurrence and development of lung cancer.

Beclin 1, a mammalian homolog of yeast Atg6, plays a central role in autophagy. It interacts with several cofactors, regulating the lipid kinase Vps-34 protein, and promotes the formation of the Beclin-1-Vps34-Vps15 core complex, inducing autophagy (Kang et al., 2011; Vega-Rubin-de-Celis, 2019; Vega-Rubin-de-Celis et al., 2020). Beclin 1 is crucial in the cross-regulation of apoptosis and autophagy in tumor cells (Menon and Dhamija, 2018). It is not only a key autophagy regulatory factor with specific interacting factors but also a potential therapeutic target for cancer (Fu et al., 2013). Studies suggest that high expression of Beclin 1 is associated with a favorable prognosis in NSCLC (Zhou et al., 2013; Zheng et al., 2018).

P62, also known as sequestosome 1, is an important autophagy receptor involved in various cellular signal transduction regulatory, oxidative stress, and autophagy processes. P62 forms a complex with ubiquitinated proteins and the LC3-II protein on the membrane of autophagic vesicles, facilitating their degradation within autolysosomes (Puissant et al., 2012).

When the level of P62 protein increases, autophagic activity is inhibited. Therefore, the amount of P62 protein can indirectly reflect the clearance level of autophagosomes, showing a negative correlation with autophagic flux (Guo et al., 2022). Conversely, if autophagic activity weakens or the autophagic system is impaired, P62 protein will continuously accumulate in the cytoplasm. In lung cancer cells, the accumulation of P62 protein may inhibit autophagic activity, preventing the normal degradation of cellular waste materials and thereby promoting the occurrence and development of lung cancer. Studies have indicated a significant correlation between the high expression of P62 and the high invasiveness and poor prognosis of lung cancer (Inoue et al., 2012; Wang et al., 2015).

To conclude, a plethora of autophagy-related genes exert an influence on the occurrence, progression, and prognostication of lung cancer. Delving into the mechanisms that underlie these genes within the framework of lung cancer provides fresh insights and strategies for the identification and treatment of this malignant condition.

### 4.3 Emerging frontiers in autophagy-related research in lung cancer

In order to investigate the evolving areas of autophagy-related research in lung cancer, an examination of the average publication year and citation bursts of specific keywords was conducted (Figures 4B, D). Keywords, such as cell proliferation, injury, migration, nanoparticles, complex, risk, invasion, progression, epithelial-mesenchymal transition, and cisplatin resistance, show the latest average publication years, suggesting current developments in the field. However, keywords, such as lung adenocarcinoma, tumor microenvironment, biology, protects, NSCLC, and cancer therapy, are experiencing citation bursts, signifying that these topics are currently at the forefront of research and likely to become future hotspots. Recent research is delving into the deep mechanisms of autophagy in lung cancer, such as autophagy in the TME, the interaction between autophagy and EMT, and the effect of autophagy on cell proliferation, migration, and invasion. Moreover, ongoing endeavors are being undertaken to explore the clinical implications of autophagy in the context of lung cancer, encompassing the pursuit of novel therapeutic approaches by targeting autophagy and enhancing resistance mechanisms, as well as identifying risk factors. The identified keywords serve as pivotal subjects in the realm of autophagy-related investigations pertaining to lung cancer, thereby indicating potential avenues for future research. The vast potential inherent in autophagy research within the domain of lung cancer portends the probability of substantial future advancements and revelations.

### 4.4 Limitations

This study comprehensively analyzed and visualized the knowledge structure and evolutionary trends of autophagy-related research in lung cancer using bibliometric methods. However, there are still some limitations. Firstly, the study only included the most commonly used and authoritative comprehensive database, WoSCC, in bibliometric research, which may lead to the omission of some literature not covered by this database. Additionally, the study only included English-language literature, potentially overlooking a few publications and studies in other languages. Furthermore, to capture the latest research trends and focus on cutting-edge topics, this study only considered literature from the past decade to avoid outdated information.

## 5 Conclusion and future perspectives

This study, to the best of our knowledge, for the first time provides a comprehensive analysis of the knowledge structure and emerging frontiers in autophagy-related research in lung cancer from 2013 to 2022 using bibliometric analysis. In summary, autophagy-related research in lung cancer is in a rapid developmental stage, with China and the United States holding core positions in this field. There is a need for strengthened international collaboration between research institutions and researchers from different countries. The research topics identified mainly revolve around five hotspots: Mechanisms influencing autophagy in lung cancer and the role of autophagy in lung cancer; Effect of autophagy on the biological behavior of lung cancer cells; Regulatory mechanisms of 2 cell death processes, autophagy and apoptosis, in lung cancer cells; Role of autophagy in lung cancer treatment and drug resistance; Role of autophagy-related genes in the occurrence and development of lung cancer. The bibliometric analysis provides a comprehensive understanding of autophagy-related research in lung cancer, serving as a valuable reference for future researchers in this field.

The future prospects of autophagy-related research in lung cancer are broad and promising. The research directions cover multiple aspects, including the optimization of treatment strategies by developing more effective drugs and new treatment combinations to utilize autophagy as a therapeutic approach for lung cancer. Additionally, in-depth studies on the molecular mechanisms of autophagy will contribute to a comprehensive understanding of its role in the onset and progression of lung cancer. Furthermore, investigating the regulation and impact of autophagy by the tumor microenvironment, along with the discovery of biomarkers associated with autophagy, may become crucial focuses in future research. Addressing the issue of drug resistance, particularly the interaction with conventional therapeutic drugs, is also anticipated to be a key topic. These research directions align with recent trends and collectively form a captivating and promising field for future autophagy-related studies in lung cancer. The trends and explorations indicate the extensive potential of autophagy research in the field of lung cancer, suggesting that significant advancements and insights may unfold in the coming years.

## Data Availability

The original contributions presented in the study are included in the article/Supplementary material, further inquiries can be directed to the corresponding author.
